# Phylogeographic analyses of an epiphytic foliose lichen show multiple dispersal events westward from the Hengduan Mountains of Yunnan into the Himalayas

**DOI:** 10.1002/ece3.9308

**Published:** 2022-09-14

**Authors:** Mei‐Xia Yang, Silke Werth, Li‐Song Wang, Christoph Scheidegger

**Affiliations:** ^1^ Biodiversity and Conservation Biology Swiss Federal Institute for Forest, Snow and Landscape Research WSL Birmensdorf Switzerland; ^2^ Institute of Plant Sciences University of Bern Bern Switzerland; ^3^ Division of Life Science and Center for Chinese Medicine The Hong Kong University of Science and Technology Hong Kong China; ^4^ Systematics, Biodiversity and Evolution of Plants, Ludwig Maximilian University Munich Munich Germany; ^5^ Key Laboratory for Plant Diversity and Biogeography of East Asia Kunming Institute of Botany, Chinese Academy of Sciences Kunming China

**Keywords:** ancestral area reconstruction, Hengduan Mountains, Himalayas, *Lobaria pindarensis*, phylogeography

## Abstract

*Lobaria pindarensis* is an endemic species of the Himalayas and the Hengduan Mountains. Little information is available on the phylogeography genetics and colonization history of this species or how its distribution patterns changed in response to the orographic history of the Himalayas and Hengduan Mountains. Based on samples covering a major part of the species' distribution range, we used 443 newly generated sequences of nine loci for molecular coalescent analyses in order to reconstruct the evolutionary history of *L. pindarensis*, and to reconstruct the species' ancestral phylogeographic distributions using Bayesian binary MCMC analyses. The results suggest that current populations originated from the Yunnan region of the Hengduan Mountains in the middle Pliocene, and that the Himalayas of Bhutan were colonized by a lineage that diverged from Yunnan ca. 2.72 Ma. The analysis additionally indicates that the Nepal and Xizang areas of the Himalayas were colonized from Yunnan as well, and that there was later a second dispersal event from Yunnan to Bhutan. We conclude that the change in climate and habitat related to the continuous uplift of the Himalayas and the Hengduan Mountains in the late Pliocene and middle Pleistocene influenced the geographic distribution pattern of *L. pindarensis*.

## Graphical abstract

Our study used 443 newly generated sequences of an endemic lichen Lobaria pindarensis in the Himalayas and Hengduan Mountains, for analyzing molecular coalescent analyses and Bayesian binary MCMC. The results suggest that current populations of *L. pindarensis* originated from the Yunnan region of the Hengduan Mountains in the middle Pliocene, and that the Himalayas of Bhutan, Xizang and Nepal were colonized by a lineage that diverged from Yunnan ca. 2.72 Ma. We conclude that the change in climate and habitat‐related to the continuous uplift of the Himalayas and the Hengduan Mountains in the late Pliocene and middle Pleistocene influenced the geographic distribution pattern of *L. pindarensis*.

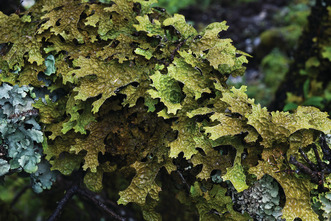

## 1 INTRODUCTION

Many regional lichen floras include endemic taxa, even though most lichen species have broad, yet often fragmented, geographic distribution ranges (Galloway, 1996). It remains unclear whether current biogeographical patterns reflect long‐distance dispersal or fragmentation of historically continuous ranges. Sexually and asexually produced lichen propagules lack special morphological adaptations for long‐distance dispersal (Dettki et al., 2000; Heinken, 1999; Sillett et al., 2000). Therefore, it has been assumed that lichens are often dispersal‐limited (Armstrong, 1990; Bailey, 1976). However, some studies indicate that lichens can disperse over very large distances, explaining the bipolar distribution patterns of some species (Fernández‐Mendoza et al., 2011; Garrido‐Benavent et al., 2018; Myllys et al., 2003; Wirtz et al., 2008). Molecular studies can help us to understand population history, determine the genetic differentiation among lichen populations, and trace past migration events (Sork & Werth, 2014; Werth et al., 2021).

**FIGURE 1:**
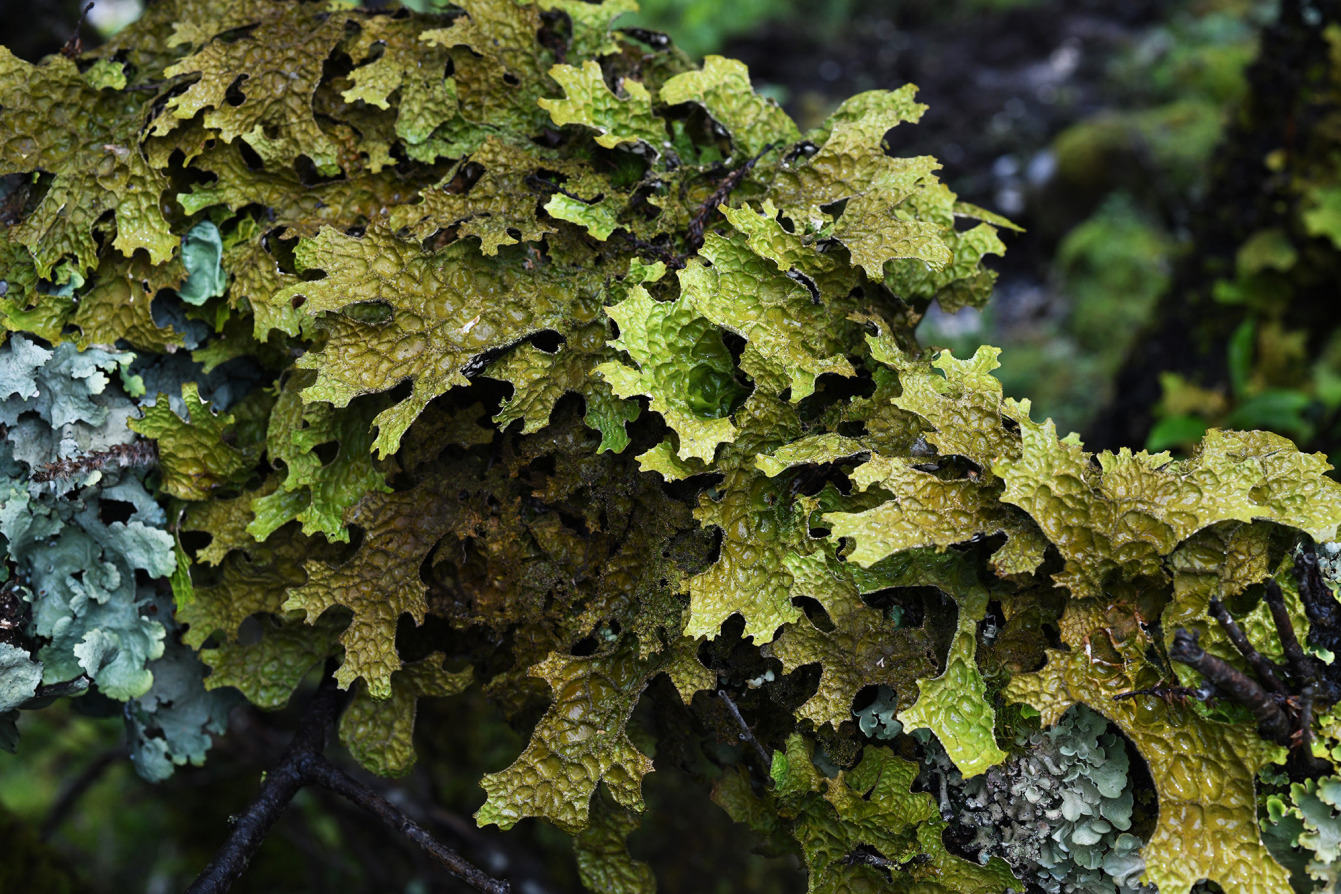
Habit of *Lobaria pindarensis*

*Lobaria pindarensis* Räsänen (Lobarioideae, Peltigeraceae) (Figure 1) is an epiphytic foliose lichen associated with a green algal photobiont. This lichen species has developed a complex reproduction strategy including propagation by fungal ascospores sexually produced in disk‐shaped fruiting bodies and by different types of asexual (symbiotic) dispersal units such as isidia, lobules, and thallus fragments (Büdel & Scheidegger, 1996; Scheidegger, 1995). The sexual cycle of the photobiont is suppressed while in symbiosis and only the mycobiont goes through sexual reproduction, resulting in the development of ascospores (Malachowski et al., 1980). *Lobaria pindarensis* grows on deciduous and coniferous trees and shrubs in subalpine forests at 2000–4150 m a.s.l. in Nepal (Devkota et al., 2017; Scheidegger et al., 2010), India (Joshi & Awasthi, 1982; Shukla et al., 2015), China (Yoshimura, 1971), and Bhutan (Aptroot & Feijen, 2002; Cornejo & Scheidegger, 2015) in the Himalayas and the Hengduan Mountains (Mts.). The two mountain systems border the Qinghai‐Tibetan Plateau (QTP), and these ecosystems are regarded to be among the most interesting study areas for exploring biodiversity (Baniya et al., 2010; Bhattarai & Vetaas, 2003; Xing & Ree, 2017). Maintenance of genetic variation is important, particularly for endangered or endemic species with a narrow distribution range, to increase their chance of long‐term survival (Holderegger & Wagner, 2008; James & Ashburner, 1997). The diversity of lichenized species in particular is very high in the Himalayas and the Hengduan Mts., and many discoveries of new species have been published recently (Aptroot & Feijen, 2002; Cornejo et al., 2018; Cornejo & Scheidegger, 2015; Devkota et al., 2017; Liu et al., 2017; Wang et al., 2017; Yang et al., 2019).

Studies of the geographic genetics of lichen‐forming fungi have led to different conclusions regarding species and geographic scale (Cassie & Piercey‐Normore, 2008; Scheidegger et al., 2012; Werth, 2010, 2011). Although several large‐scale studies have been conducted at an intercontinental scale or within European ecosystems (Buschbom, 2007; Dal Grande et al., 2010; Fernández‐Mendoza et al., 2011; Geml et al., 2010; Otalora et al., 2015; Walser et al., 2003, 2005; Werth et al., 2021), very few population genetic studies of *Lobaria* species have been carried out in the Himalayas and Hengduan Mts. Devkota, Chaudhary, et al. (2019) and Devkota, Dymytrova, et al. (2019) studied the genetic diversity of *L. pindarensis* populations throughout the species' distribution range in Nepal, applying 17 fungus‐specific and nine alga‐specific microsatellite loci, and showed that genetic diversity, allelic richness and gene pool composition were significantly influenced by elevation.

Here, we investigated the genetic variation at nine nuclear loci in the mycobiont of the epiphytic lichen *Lobaria pindarensis* from the Hengduan Mts. and the Himalayas. We aimed to identify the species' region of origin, evolutionary history, and range expansion by combining information related to geographic and climatic events in the Himalayas and the Hengduan Mts. We present data on the phylogeography of this endemic lichen species in the Himalayas and the Hengduan Mts., which can be used as a reference for future research on the phylogeography of lichens.

## 2 MATERIALS AND METHODS

### 2.1 Study area

A total of 53 individuals were collected during various excursions. In 2017, we (CS, LS and MY) made a reconnaissance visit to Yunnan province in China, the central distribution range of *Lobaria pindarensis* in the Hengduan Mts. In 2017, CS made a reconnaissance visit to Bhutan and in 2019, LS and MY collected in eastern Xizang, the central distribution range of *L. pindarensis* in the Himalayan Mts. In addition, three specimens from Nepal were collected by Devkota and Scheidegger and discussed in a previous study (Cornejo & Scheidegger, 2018). The distribution of *L. pindarensis* along the elevation gradient of 2300–4140 m was studied in four subregions (Yunnan, Xizang, Nepal, Bhutan), based on the entire dataset of 53 geographic specimens and the elevation span of individuals from Nepal Devkota, Chaudhary, et al. (2019) and Devkota, Dymytrova, et al. (2019).

### 2.2 Sampling of specimens for genetic analyses

Thalli of *Lobaria pindarensis* were sampled during 2017–2019. In total, 53 thallus fragments of *L. pindarensis* were sampled from different collection sites (Table S1), and two samples of *L. devkotae* M. X. Yang & Scheid. were used as the outgroup since *L. devkotae* is the sister clade of *L. pindarensis* according to the phylogenetic study of green‐algal *Lobaria* in the Himalayas and the Hengduan Mountains (Yang et al., 2022). Samples were collected in an envelope and air‐dried. To ensure complete drying, the envelopes containing samples also contained dried silica gel. After the expeditions, the dried specimens were deposited in the frozen collection (−20°C) at the Swiss Federal Institute for Forest, Snow and Landscape Research WSL, Switzerland. Moreover, sets of specimens from the Xizang collection were deposited in the herbarium of the Kunming Institute of Botany (KUN‐L), Chinese Academy of Sciences.

### 2.3 DNA extraction, PCR amplification, and sequencing

Genomic DNA was extracted from freshly collected and frozen herbarium specimens. About 15 mg of visually uncontaminated lichen thallus was used from each specimen for molecular analyses. Frozen lichen samples were lyophilized and disrupted with a 3 mm stainless steel bead in a Retsch MM2000 mill (Düsseldorf, Germany) for 2 min at 30 Hz. Genomic DNA was extracted using the Qiagen DNeasy Plant Kit (QIAGEN, Hilden, Germany), following the manufacturer's Plant Tissue Mini protocol.

To increase the phylogenetic information, we employed six loci (Lpi02, Lpi09, Lpi10, Lpi11, Lpi14, and Lpi19) that contain Simple Sequence Repeats (SSRs or microsatellites) (Cornejo et al., 2018; Devkota et al., 2014). Additionally, two coding regions (partial sequences of two single‐copy loci: the RNA polymerase II gene, *RPB2*, and the translation elongation factor‐1α gene, *EF‐1α*) and the noncoding nuclear ribosomal internal transcribed spacers, nrITS, enhanced our dataset to nine loci.

The DNA isolation of all specimens, the PCR and the cycle sequencing of the *EF‐1α*, ITS and *RPB2* loci were performed as described by Cornejo and Scheidegger (2015). The PCRs used for the amplification of the SSRs containing loci followed the conditions described by Devkota et al. (2014). These amplicons were labeled with the M13‐technique used in the BigDye® Direct Sequencing Kit (Thermo Fisher Scientific, Waltham, Massachusetts, USA). The PCR products were sent to Microsynth AG (Balgach, Switzerland) for sequencing with the same primers as used for PCR amplifications. Microsatellite sequences of Lpi02, Lpi09, Lpi10, Lpi11, Lpi14, and Lpi19 were manually excised during alignment procedures in a way that only flanking regions were left. Nevertheless, all but Lpi02, Lpi11, and RPB2 datasets still contained ambiguously aligned regions, which were processed with the software Gblocks 0.91b on the freely available platform phylogeny.fr (Castresana, 2000; Dereeper et al., 2008, 2010; Devier et al., 2010) enabling smaller final blocks, gaps on the final blocks and fewer strict flanking positions.

### 2.4 Molecular phylogeny of *Lobaria pindarensis*

Phylogenetic relationships were reconstructed using sequences of the nine loci (ITS, *RPB2*, *EF‐1α*, Lpi02, Lpi09, Lpi10, Lpi11, Lpi14, and Lpi19) which were compiled for different analytical purposes. All sequences were aligned and edited using Geneious version 7.1.9 (https://www.geneious.com). All newly produced sequences were checked using the BLASTN suite of the National Center for Biotechnology Information (NCBI) website (http://www.ncbi.nlm.nih.gov/BLAST/) to verify their close relatives and preclude potential contaminants (Ye et al., 2006). Matrices were aligned with MAFFT (version 7) web service (http://mafft.cbrc.jp/alignment/server/index.html) (Katoh et al., 2019). Specimens used in this study, along with voucher information from the GenBank accession numbers, are listed in Table S2. Phylogenetic and molecular evolutionary analyses were conducted using MEGA version 6 (Tamura et al., 2013). The ambiguously aligned regions were arranged manually for the phylogenetic analyses. The gene fragments were combined using Geneious 7.1.9 for phylogenetic analysis, on the premise that no well‐supported (BS > 70%, Nuhn et al., 2013) conflict was detected. Maximum likelihood (ML) and Bayesian inference (BI) analyses were performed using RaxML v. 7.2.6 (Stamatakis, 2006) and MrBayes 3.1.2 (Ronquist & Huelsenbeck, 2003), respectively. We calculated the nine gene‐trees under a maximum likelihood criterion. Models of the DNA sequence evolution for each locus were selected with the program jModelTest 3.7 (Posada, 2008) by using the Akaike information criterion (Akaike, 1973). Maximum likelihood analyses were conducted using RaxML v. 7.2.6 and run on the ATGC bioinformatics platform (atgc‐montpellier.fr), which enables the application of different substitution models. The bootstrap values were calculated with 1000 replicates. The best option was used, which estimates the phylogeny based on two different methods and returns the better of the two solutions. For BI analyses, four Markov Chain Monte Carlo (MCMC) chains were run simultaneously for 20 million generations with trees sampled every 100 generations. We considered the sampling of the posterior distribution to be adequate when the average standard deviation of split frequencies was <0.01. Chain convergence was determined by checking the effective sampling size (ESS > 200) in Tracer 1.6 (Rambaut et al., 2014). By omitting the first 25% of trees as burn‐ins using the “sump” and “sumt” commands, a majority rule consensus tree was generated. Clades were judged using both ML Bootstrap (MLB ≥70%) and Bayesian posterior probabilities (BPP ≥0.9). The tree files were visualized with FigTree 1.4.3 (Rambaut et al., 2014) and edited using Adobe Photoshop CS6 (Adobe Systems Incorporated, San Jose, CA, USA).

### 2.5 Generating a time‐calibrated tree of *Lobaria pindarensis*

We used a Bayesian method, implemented in the programs BEAUti and BEAST (both version 1.8.4; Drummond et al., 2012), to estimate a time‐tree. Secondary calibrations (calibrations based on the results of previous molecular dating studies) (Cornejo et al., 2018) are applied in divergence time analyses. Bayesian inference (BI) based on Markov chain Monte Carlo methods (Yang & Rannala, 1997) was performed using MrBayes 3.1.2 (Ronquist & Huelsenbeck, 2003). The optimal model of molecular evolution and gamma rate heterogeneity was determined as implemented in jModelTest (Posada, 2008) by using Akaike Information Criterion (AIC). The Markov chain Monte Carlo (MCMC) algorithm was run for 20,000,000 generations with one cold and three heated chains, staring from random trees. Runs were repeated twice. We selected a Yule speciation model for the species‐tree prior, and the population size model was set to piecewise linear and constant root. Clock and tree parameters were linked across partitions and default values were used for the remaining priors. We ran MCMC analyses of 25 million generations with a burn‐in of 10% for each run. We assessed the effective sample sizes (ESS) of parameters of interest with Tracer version 1.6 (Rambaut et al., 2014) to ensure that sample sizes were all greater than 200. The maximum clade credibility tree, including posterior probabilities of branches, was computed with TreeAnnotator version 1.8.4 (included in the BEAST 1.8.4 package) from the sampled trees after exclusion of the burn‐in. The best tree was visualized in FigTree (Version 1.4.3) after exclusion of the burn‐in.

### 2.6 Ancestral area reconstruction

The distribution range of *Lobaria pindarensis* was divided into four biogeographical subregions, as follows: A (Yunnan), B (Bhutan), C (Xizang), and D (Nepal). The ancestral geographic ranges at each node were reconstructed using Bayesian Binary MCMC (BBM) analysis in the program Reconstruct Ancestral States in Phylogenies version 4.0 (RASP; Yu et al., 2015). In RASP, the BBM method inputs a posterior distribution of Bayesian inference, in this study the consensus tree from BEAST, to reconstruct the possible ancestral distributions of given nodes via a hierarchical Bayesian approach (Ronquist & Huelsenbeck, 2003). Using the R package *BioGeoBEARS*, we collapsed the phylogeny to a monophyletic population tree (Matzke, 2013) whose tips are monophyletic populations instead of samples. Outgroups were excluded, as the input phylogeny should include only monophyletic groups (Matzke, 2013) without an outgroup (Yu et al., 2015). The maximum number of areas occupied at each node was set to four. To account for phylogenetic uncertainty, 5 million generations of the MCMC chains were run, with sampling every 100 generations.

## 3 RESULTS

### 3.1 Geographic genetics and divergence time estimates

For the genetic analyses of *Lobaria pindarensis*, separated partitions of the nine loci, which contained 2900 unambiguously aligned nucleotide characters, were loaded in BEAUti. Two specimens of *L. devkotae* were included as an outgroup. In total, we generated 443 new mycobiont‐specific sequences (55 ITS, 33 RPB2, 33 *EF‐1α*, 55 Lpi02, 54 Lpi09, 55 Lpi10, 48 Lpi11, 55 Lpi14, and 55 Lpi19). For GenBank accession numbers, see Table S2.

**FIGURE 2:**
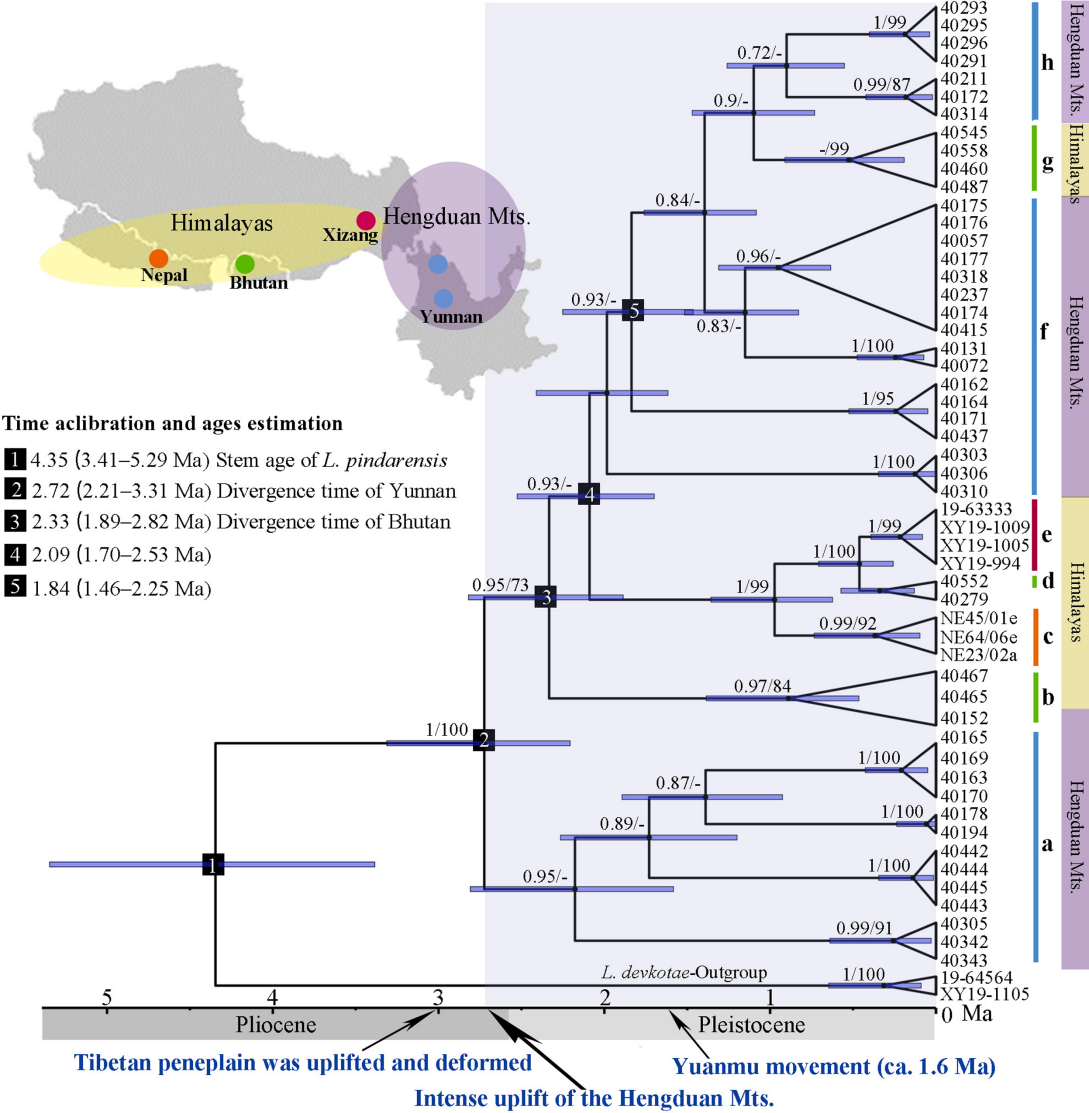
Time‐calibrated phylogenetic tree derived from BEAST based on nine loci (ITS, EF‐1α, RPB2, Lpi02, Lpi09, Lpi10, Lpi11, Lppi14, and Lpi19) for *Lobaria pindarensis*. Bayesian divergence time estimates of the main nodes are listed on the left. ML bootstrap support values/Bayesian posterior probabilities greater than 70%/0.9 are indicated. Blue bars represent the 95% highest posterior density intervals for node (mean) ages. With different colors indicating the distribution of the major clades. The color bands on the right side: Purple (Hengduan mts.) and yellow (Himalayas). The outgroup‐species L. devkotae was used to root the tree. The major geographic events related to the uplift of the Himalayas and the Hengduan mts. Are indicated at the bottom.

The phylogenetic analysis revealed a well‐supported topology in which populations generally clustered according to their geographic proximity (Figure 2). The phylogenetic tree indicated that the clade a from Yunnan, firstly branched off at node 2 to become a sister lineage for the rest of the lineages (Figure 2). All interior nodes of each region and subregion had bootstrap support/posterior probabilities higher than 70%/0.9. According to the BEAST analyses, the stem age of *L. pindarensis* was 4.35 million years ago [Ma] (3.41–5.29 Ma; 95% highest posterior density, HPD). The divergence time estimates showed that the Himalayan populations started to diverge from the Hengduan population ca. 2.72 Ma (2.21–3.31 Ma; 95% HPD). All subregions from the Himalayas and the Hengduan Mts. form a monophyletic clade that diverged from Clade a (Yunnan lineage) within the last 2.72 million years [Myr] (Figure 2).

### 3.2 Ancestral area reconstruction

**FIGURE 3:**
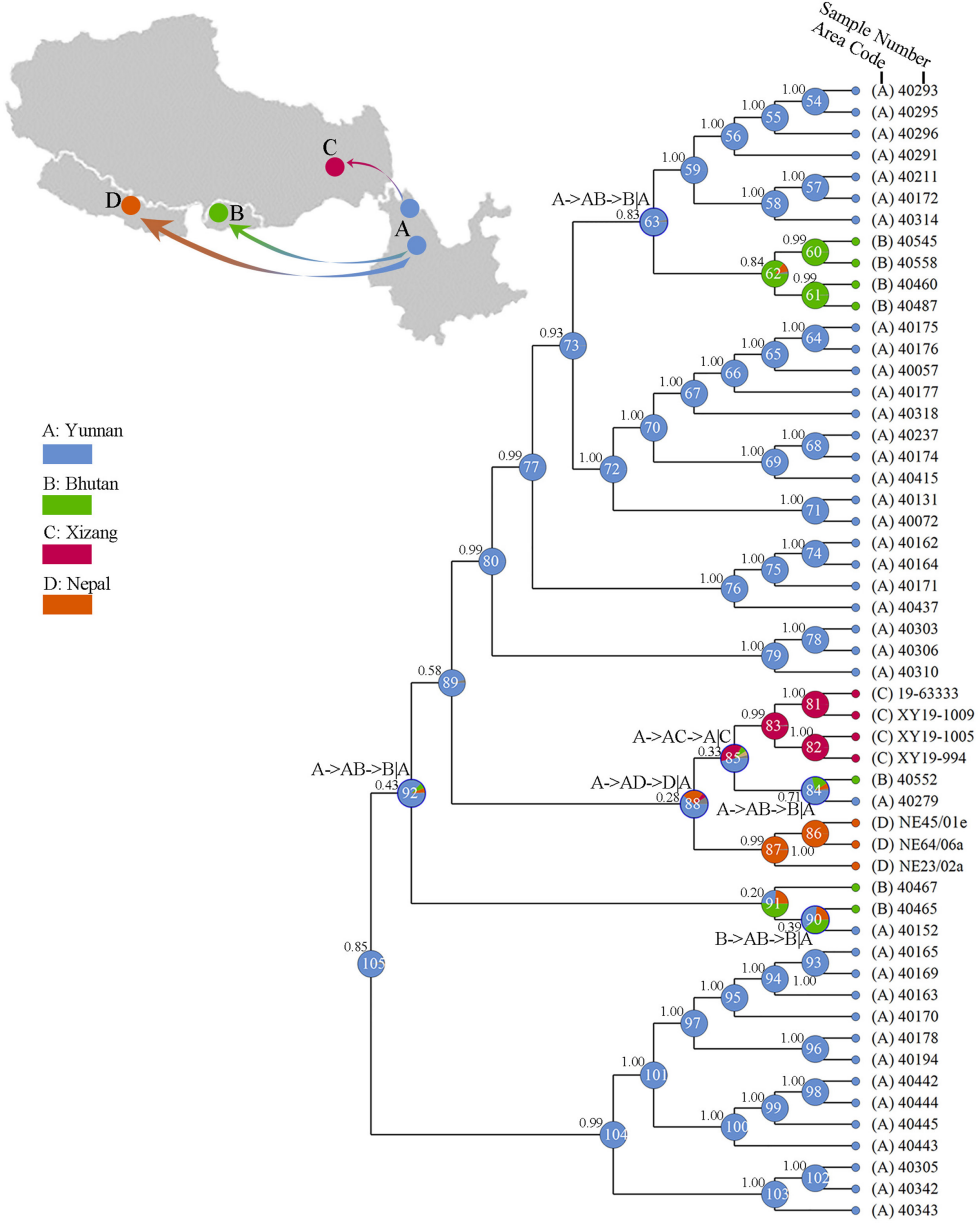
Ancestral area reconstruction for *Lobaria pindarensis* based on Bayesian binary MCMC conducted in RASP. Letters on the map represent the current distribution areas of *L. pindarensis*. Definitions and abbreviations: A (Yunnan), B (Bhutan), C (Xizang) and D (Nepal). The pie charts show the probabilities of ancestral area reconstructions.

The ancestral area reconstruction analyses suggested that the first range expansion of *Lobaria pindarensis* occurred in the Pliocene (Figure 3) out of Yunnan, which is considered the ancestral area of this species, since the ancestral range for node 104 was Yunnan in the BBM analysis, with 0.99 posterior probability. The postulated ancestral range of the entire lineage (node 105) was Yunnan. Putting together nodes 105, 92 and 89, ancestral area reconstruction suggests an early migration from Yunnan to Bhutan within the last 2.72 Myr (2.21–3.31 Ma; 95% HPD). Subsequently, the species migrated from Yunnan to Nepal and Xizang around 2.33 Ma (1.98–2.82 Ma; 95% HPD). Moreover, there was another migration event from Yunnan to Bhutan within the last 1.84 Myr (1.46–2.25 Ma; 95% HPD). Thus, the results indicated a colonization scenario in which *L. pindarensis* originated in the Hengduan Mts. and migrated westward to the Himalayas through several migration events.

## 4 DISCUSSION

*Lobaria pindarensis* is distributed in subalpine areas between 2300 and 4140 m a.s.l. in the Himalayas and the Hengduan Mts. Throughout the wide east–west distribution of the species, we found high levels of genetic diversity and differentiation, which indicate a complex process of westward range expansion during the species' evolutionary history since its origin 4.35 Ma.

The biogeographical analyses of *Lobaria pindarensis* point to a westward range expansion in the early Pleistocene (ca. 2.76 Ma). The ancestral area reconstruction analyses suggest that the Hengduan Mts. are the origin of *L. pindarensis* (Figure 3). From Yunnan, *L. pindarensis* migrated to the eastern Himalayas (Bhutan) in the early Pleistocene and after 2.09 Myr to the central and western Himalayas and to northeastern regions of Xizang. At a later stage, just over 1 Ma, there was another dispersal event from the Hengduan Mts. to Bhutan.

The Qinghai‐Tibetan Plateau (QTP) has undergone uplift and peneplanation since the beginning of the Tertiary (Xu & Zhang, 1981). The plateau was probably at about 1000 m a.s.l. during the Pliocene (Cao et al., 1981; Mao et al., 2021). At that time, the climate of southern Xizang was relatively warm and wet, but the northern regions were more arid and could not support glaciers (Spicer, 2017; Xu, 1992). Compared with other mountain ranges bordering the QTP, the Hengduan Mts. are younger, having uplifted over the last 8 Myr, and they showed a very high species diversification rate during this period (Xing & Ree, 2017). During the continuing uplift the basins within the QTP reached 3000 m a.s.l., and the mountains reached ∼4000–4500 m a.s.l. at the end of the Early Pleistocene (Zheng et al., 2002). These elevation ranges may have enabled species migration in what was likely an ecologically diverse region, ranging from the warm and moist Hengduan Mts. to the cold and moist Himalayas. Our recent research (Yang et al., 2022) of green‐algal *Lobaria* also showed that the low‐altitude regions and mountain gorges of the Hengduan Mountains and the Himalayas were important refugia during the glacial periods. Species in this region responded to environmental changes by migrating north and south along the Hengduan Mountains, corresponding to a change in altitude. After migration and colonization, populations were isolated for a long time in glacial refugia formed by Pleistocene glaciers, which promoted population differentiation and speciation.

Our data and analyses clarify the evolutionary history of *Lobaria pindarensis*, which is consistent with the history of the Himalayas and the Hengduan Mts. The Tibetan peneplain uplift and deformation, the intensive uplift of the Hengduan Mts., and the Yuanmu movement in the Himalayas fit with our model of *L. pindarensis* biogeography (Figure 3). The time estimates show that the migration of *L. pindarensis* appeared during the Pliocene and that the species diversification and migration largely occurred ca. 3.31–2.21 Myr out of Yunnan (Hengduan Mts.) to Bhutan (Himalayas). Our findings on pattern and processes of *L. pindarensis* differentiation parallel other plants and animal species in the Himalayas and the Hengduan Mts. regions (Cao et al., 2012; Cun & Wang, 2010; Huang et al., 2013). Our studies also confirmed a distinct intraspecific differentiation in *L. pindarensis* in these regions that served as a diversification and divergence center which was due to the intense uplift of the Hengduan Mts at the end of the Pliocene (Meng et al., 2017; Mosbrugger et al., 2018) and which served as a refugium for *L. pindarensis* during the Quaternary Ice Ages. The uplift (Yuanmu movement) during the latter part of the Early Pleistocene helped to create the higher mountains of the Himalayas, allowing small ice caps or piedmont glaciers to form north of the Himalayas in regions such as northwest Xizang. During this time, the elevation of the lake basin floors was ∼2000–2500 m a.s.l. The climate had become substantially colder (Zheng et al., 2002), which may have created additional forest habitat enabling increased connectivity between the Hengduan Mts. and the Himalayas.

Based on our field investigations, it is clear that infrastructure construction and forest management activities in these regions considerably reduce and threaten *L. pindarensis* habitats in areas that have recently been under intensive development. Further, the use of *L. pindarensis* in traditional medicine and food by cultures across the Himalayas and Hengduan Mts. (Yang et al., 2021) contributes to its sensitive/threatened status. Effective conservation strategies for the forest landscapes of the Himalayas and Hengduan Mts. must therefore ensure that these unique cradles of biodiversity do not turn into graves for biodiversity (Rangel et al., 2018), including for lichens like *L. pindarensis*.

## AUTHOR CONTRIBUTIONS

**Meixia Yang:** Conceptualization (lead); data curation (lead); formal analysis (lead); investigation (equal); methodology (lead); resources (lead); software (lead); validation (equal); visualization (lead); writing – original draft (lead); writing – review and editing (lead). **Silke Werth:** Conceptualization (equal); methodology (equal); supervision (equal); visualization (equal); writing – review and editing (equal). **Lisong Wang:** Funding acquisition (lead); investigation (lead); resources (equal); supervision (equal); writing – review and editing (equal). **Christoph Scheidegger:** Conceptualization (lead); data curation (equal); funding acquisition (lead); investigation (equal); methodology (equal); project administration (lead); supervision (lead); visualization (lead); writing – review and editing (lead).

## CONFLICT OF INTEREST

The authors declare no conflicts of interest.

## Supporting information


Tables S1–S2

## Data Availability

Sequencing data: GenBank accession numbers and voucher information are listed in Table S1 and S2.
