# Supplementary material for: Phylogeographic analyses of an epiphytic foliose lichen show multiple dispersal events westward from the Hengduan Mountains of Yunnan into the Himalayas
Source: Ecol Evol. 2022 Sep 14;12(9):e9308. doi: 10.1002/ece3.9308 (PMC9475131; doi:10.1002/ece3.9308)
Supplement: Supplementary file 1 — Tables S1–S2 [file ECE3-12-e9308-s001.docx]

**Supplemental Information for:**

**Phylogeographic analyses of an epiphytic foliose**

**lichen show multiple dispersal events westwards from the**

**Hengduan Mountains of Yunnan into the Himalayas**

Mei-Xia Yang, Silke Werth, Li-Song Wang, Christoph Scheidegger

**TABLE S1** Origin and collectors of specimens used in this study.

| **Collection site** | **Isolate** | **Herbarium** | **Collector(s)** |
| --- | --- | --- | --- |
| Bhutan, Paro District, Taktshang (Tiger’s Nest) | 40460, 40465, 40467, 40487, 40545, 40552, 40558 | Scheidegger | C. Scheidegger |
| Nepal | NE23/02a, NE45/01e, NE64/06a | TUCH | S. Devkota and C. Scheidegger |
| Xizang, Bomi County, on route from Ranwu to Bomi | 19-63333 | KUN-L | L. S. Wang and M. X. Yang |
| Xizang, Bayi District, on route from Bomi to Lulang | 19-64564 | KUN-L | L. S. Wang and M. X. Yang |
| Xizang, Prope Bomi County | XY19-994, XY19-1009 | KUN-L | L. S. Wang and X. Y. Wang |
| Xizang, Bomi County, Gu Village, on route from Bomi to Linzhi | XY19-1005 | KUN-L | L. S. Wang and X. Y. Wang |
| Xizang, Bayi District, Lulang Town | XY19-1105 | KUN-L | L. S. Wang and X. Y. Yang |
| Yunnan, Lijiang City, Laojunshan Mt., Sanxuan Lake | 40057, 40072, 40131, 40152 | Scheidegger | C. Scheidegger, L. S. Wang and M. X. Yang |
| Yunnan, Lijiang City, alpine botanical garden | 40162, 40164, 40163, 40165, 40169, 40170, 40171, 40172, 40174, 40175, 40176, 40177, 40178, 40194, 40211, 40237 | Scheidegger | C. Scheidegger, L. S. Wang and M. X. Yang |
| Yunnan, Shaggrila City, Tianbao Mt., coniferous forest | 40291, 40293, 40295, 40296, 40303, 40305, 40306, 40310, 40314, 40318, 40342, 40343, 40415, 40437, 40442, 40443, 40444, 40445 | Scheidegger | C. Scheidegger, L. S. Wang and M. X. Yang |

Scheidegger: private herbarium, Swiss Federal Institute for Forest, Snow and Landscape Research WSL, Switzerland.

KUN-L: Lichen Herbarium of the Kunming Institute of Botany, Chinese Academy of Sciences, China.

TUCH: Tribhuvan University Central Herbarium, Tribhuvan University, Kathmandu Nepal.

**TABLE S2** Overview of 53 *Lobaria pindarensis* and 2 *L. devkotae*specimens, with the respective voucher information from Genbank. Sequences newly obtained in this study are indicated in bold.

| **Specimen ID** | **GenBank number** | | | | | | | | |
| --- | --- | --- | --- | --- | --- | --- | --- | --- | --- |
|  | **ITS** | ***EF-1α*** | ***RPB2*** | **Lpi02** | **Lpi09** | **Lpi10** | **Lpi11** | **Lpi14** | **Lpi19** |
| 40293 | **MZ724590** | **MZ772508** |  | **MZ772579** | **MZ772631** | **MZ772682** | **MZ772732** | **MZ772779** | **MZ772831** |
| 40295 | **MZ724591** | **MZ772509** | **MZ772537** | **MZ772580** | **MZ772632** | **MZ772683** | **MZ772733** | **MZ772780** | **MZ772832** |
| 40296 | **MZ724592** | **MZ772510** |  | **MZ772581** | **MZ772633** | **MZ772684** |  | **MZ772781** | **MZ772833** |
| 40291 | **MZ724589** |  | **MZ772536** | **MZ772578** | **MZ772630** | **MZ772681** | **MZ772731** | **MZ772778** | **MZ772830** |
| 40211 | **MZ724586** | **MZ772506** | **MZ772534** | **MZ772575** | **MZ772627** | **MZ772678** |  | **MZ772775** | **MZ772827** |
| 40172 | **MZ724579** |  |  | **MZ772568** | **MZ772620** | **MZ772671** | **MZ772722** | **MZ772768** | **MZ772820** |
| 40314 | **MZ724597** | **MZ772513** | **MZ772540** | **MZ772586** | **MZ772638** | **MZ772689** | **MZ772738** | **MZ772786** | **MZ772838** |
| 40545 | **MZ724611** |  |  | **MZ772600** | **MZ772652** | **MZ772703** | **MZ772750** | **MZ772800** | **MZ772852** |
| 40558 | **MZ724613** |  |  | **MZ772602** | **MZ772654** | **MZ772705** | **MZ772751** | **MZ772802** | **MZ772854** |
| 40460 | **MZ724607** | **MZ772519** | **MZ772546** | **MZ772596** | **MZ772648** | **MZ772699** | **MZ772747** | **MZ772796** | **MZ772848** |
| 40487 | **MZ724610** | **MZ772521** | **MZ772548** | **MZ772599** | **MZ772651** | **MZ772702** | **MZ772749** | **MZ772799** | **MZ772851** |
| 40175 | **MZ724581** | **MZ772504** | **MZ772532** | **MZ772570** | **MZ772622** | **MZ772673** | **MZ772724** | **MZ772770** | **MZ772822** |
| 40176 | **MZ724582** | **MZ772505** | **MZ772533** | **MZ772571** | **MZ772623** | **MZ772674** | **MZ772725** | **MZ772771** | **MZ772823** |
| 40057 | **MZ724568** |  | **MZ724568** | **MZ772557** | **MZ772609** | **MZ772660** | **MZ772712** | **MZ772757** | **MZ772809** |
| 40177 | **MZ724583** |  |  | **MZ772572** | **MZ772624** | **MZ772675** | **MZ772726** | **MZ772772** | **MZ772824** |
| 40318 | **MZ724598** | **MZ772514** | **MZ772541** | **MZ772587** | **MZ772639** | **MZ772690** | **MZ772739** | **MZ772787** | **MZ772839** |
| 40237 | **MZ724587** |  |  | **MZ772576** | **MZ772628** | **MZ772679** | **MZ772729** | **MZ772776** | **MZ772828** |
| 40174 | **MZ724580** |  |  | **MZ772569** | **MZ772621** | **MZ772672** | **MZ772723** | **MZ772769** | **MZ772821** |
| 40415 | **MZ724601** |  |  | **MZ772590** | **MZ772642** | **MZ772693** | **MZ772742** | **MZ772790** | **MZ772842** |
| 40131 | **MZ724570** | **MZ772499** | **MZ772527** | **MZ772559** | **MZ772611** | **MZ772662** | **MZ772714** | **MZ772759** | **MZ772811** |
| 40072 | **MZ724569** | **MZ772498** | **MZ772526** | **MZ772558** | **MZ772610** | **MZ772661** | **MZ772713** | **MZ772758** | **MZ772810** |
| 40162 | **MZ724572** |  |  | **MZ772561** | **MZ772613** | **MZ772664** | **MZ772716** | **MZ772761** | **MZ772813** |
| 40164 | **MZ724574** |  |  | **MZ772563** | **MZ772615** | **MZ772666** | **MZ772718** | **MZ772763** | **MZ772815** |
| 40171 | **MZ724578** |  |  | **MZ772567** | **MZ772619** | **MZ772670** | **MZ772721** | **MZ772767** | **MZ772819** |
| 40437 | **MZ724602** |  |  | **MZ772591** | **MZ772643** | **MZ772694** | **MZ772743** | **MZ772791** | **MZ772843** |
| 40303 | **MZ724593** | **MZ772511** | **MZ772538** | **MZ772582** | **MZ772634** | **MZ772685** | **MZ772734** | **MZ772782** | **MZ772834** |
| 40306 | **MZ724595** |  |  | **MZ772584** | **MZ772636** | **MZ772687** | **MZ772736** | **MZ772784** | **MZ772836** |
| 40310 | **MZ724596** |  |  | **MZ772585** | **MZ772637** | **MZ772688** | **MZ772737** | **MZ772785** | **MZ772837** |
| 19-63333 | **MZ724614** | **MZ772497** | **MZ772525** | **MZ772603** | **MZ772655** | **MZ772706** | **MZ772752** | **MZ772803** | **MZ772855** |
| XY19-1009 | **MZ724617** | **MZ772524** | **MZ772552** | **MZ772555** | **MZ772607** | **MZ772658** | **MZ772710** | **MZ772755** | **MZ772807** |
| XY19-1005 | **MZ724616** | **MZ772523** | **MZ772551** | **MZ772554** | **MZ772606** | **MZ772657** | **MZ772709** | **MZ772756** | **MZ772806** |
| XY19-994 | **MZ724615** | **MZ772522** | **MZ772550** | **MZ772553** | **MZ772605** | **MZ772656** | **MZ772708** | **MZ772753** | **MZ772805** |
| 40552 | **MZ724612** |  | **MZ772549** | **MZ772601** | **MZ772653** | **MZ772704** |  | **MZ772801** | **MZ772853** |
| 40279 | **MZ724588** | **MZ772507** | **MZ772535** | **MZ772577** | **MZ772629** | **MZ772680** | **MZ772730** | **MZ772777** | **MZ772829** |
| NE45/01e | MF151244 | MF151197 | MF151708 | MF151302 | MF151382 | MF151448 | MF151518 | MF151606 | MF151674 |
| NE64/06a | MF151246 | MF151198 | MF151704 | MF151303 | MF151383 | MF151449 | MF151519 | MF151607 | MF151675 |
| NE23/02a | KC494086 | KC602010 | KC602556 | MF151298 | MF151378 | MF151450 | MF151514 | MF151603 | MF151670 |
| 40467 | **MZ724609** |  |  | **MZ772598** | **MZ772650** | **MZ772701** | **MZ772748** | **MZ772798** | **MZ772850** |
| 40465 | **MZ724608** | **MZ772520** | **MZ772547** | **MZ772597** | **MZ772649** | **MZ772700** | **MZ772746** | **MZ772797** | **MZ772849** |
| 40152 | **MZ724571** | **MZ772500** | **MZ772528** | **MZ772560** | **MZ772612** | **MZ772663** | **MZ772715** | **MZ772760** | **MZ772812** |
| 40165 | **MZ724575** | **MZ772502** | **MZ772530** | **MZ772564** | **MZ772616** | **MZ772667** | **MZ772719** | **MZ772764** | **MZ772816** |
| 40169 | **MZ724576** |  |  | **MZ772565** | **MZ772617** | **MZ772668** | **MZ772720** | **MZ772765** | **MZ772817** |
| 40163 | **MZ724573** | **MZ772501** | **MZ772529** | **MZ772562** | **MZ772614** | **MZ772665** | **MZ772717** | **MZ772762** | **MZ772814** |
| 40170 | **MZ724577** | **MZ772503** | **MZ772531** | **MZ772566** | **MZ772618** | **MZ772669** |  | **MZ772766** | **MZ772818** |
| 40178 | **MZ724584** |  |  | **MZ772573** | **MZ772625** | **MZ772676** | **MZ772727** | **MZ772773** | **MZ772825** |
| 40194 | **MZ724585** |  |  | **MZ772574** | **MZ772626** | **MZ772677** | **MZ772728** | **MZ772774** | **MZ772826** |
| 40442 | **MZ724603** | **MZ772516** | **MZ772543** | **MZ772592** | **MZ772644** | **MZ772695** | **MZ772744** | **MZ772792** | **MZ772844** |
| 40444 | **MZ724605** | **MZ772517** | **MZ772544** | **MZ772594** | **MZ772646** | **MZ772697** | **MZ772745** | **MZ772794** | **MZ772846** |
| 40445 | **MZ724606** | **MZ772518** | **MZ772545** | **MZ772595** | **MZ772647** | **MZ772698** | **MZ772746** | **MZ772795** | **MZ772847** |
| 40443 | **MZ724604** |  |  | **MZ772593** | **MZ772645** | **MZ772696** |  | **MZ772793** | **MZ772845** |
| 40305 | **MZ724594** | **MZ772512** | **MZ772539** | **MZ772583** | **MZ772635** | **MZ772686** | **MZ772735** | **MZ772783** | **MZ772835** |
| 40342 | **MZ724599** |  |  | **MZ772588** | **MZ772640** | **MZ772691** | **MZ772740** | **MZ772788** | **MZ772840** |
| 40343 | **MZ724600** | **MZ772515** | **MZ772542** | **MZ772589** | **MZ772641** | **MZ772692** | **MZ772741** | **MZ772789** | **MZ772841** |
| 19-64564 | MZ724567 | MW393963 | MW393999 | **MZ772604** |  | **MZ772707** |  | **MZ772804** | **MZ772856** |
| XY19-1105 | MZ724618 | MW393969 | MW394005 | **MZ772556** | **MZ772608** | **MZ772659** | **MZ772711** | **MZ772756** | **MZ772808** |
